# First-Line Treatments and Management of Metastatic Renal Cell Carcinoma Patients: An Italian Interdisciplinary Uro-Oncologic Group Algorithm

**DOI:** 10.3390/cells13110961

**Published:** 2024-06-02

**Authors:** Francesco Bloise, Fiorella Manfredi, Luca Zatteri, Giovanni Dima, Chiara Carli, Rosanna Di Vita, Maria Olivieri, Enrico Sammarco, Marco Ferrari, Alessia Salfi, Adele Bonato, Debora Serafin, Natalia Coccia, Laura Doni, Luca Galli, Michele Sisani, Giandomenico Roviello, Martina Catalano, Federico Paolieri

**Affiliations:** 1 Medical Oncology Unit, San Donato Hospital, Azienda Toscana Sud Est, 52100 Arezzo, Italy; francesco.bloise@uslsudest.toscana.it (F.B.); michele.sisani@uslsudest.toscana.it (M.S.); 2 Medical Oncology Unit, Sant’Andrea Hospital, Azienda Sanitaria Locale 5 Spezzino, 19124 La Spezia, Italy; manfredifiorella@gmail.com; 3 Medical Oncology Unit 2, Santa Chiara Hospital, Azienda Ospedaliero-Universitaria Pisana, 56124 Pisa, Italy; lucazatteri@alice.it (L.Z.); dima_giovanni@libero.it (G.D.); chiaracarli25@gmail.com (C.C.); rosanna.dv84@gmail.com (R.D.V.); mariaolivieri1995@gmail.com (M.O.); marco.ferrari0201@gmail.com (M.F.); alessiasalfi@gmail.com (A.S.); adelebonato@gmail.com (A.B.); serafindebora@gmail.com (D.S.); nat89@tiscali.it (N.C.); lugal71@yahoo.it (L.G.); 4 Medical Oncology Unit, Livorno Hospital, Azienda Toscana Nord Ovest, 57124 Livorno, Italy; enricosammarco1992@gmail.com; 5 Clinical Oncology Unit, Careggi University Hospital, 50134 Florence, Italy; donila@aou-careggi.toscana.it; 6 Department of Health Sciences, University of Florence, 50134 Florence, Italy; giandomenico.roviello@unifi.it; 7 Department of Oncology, Hospital of Prato, Azienda USL Toscana Centro, 59100 Prato, Italy; federico.paolieri@gmail.com

**Keywords:** metastatic renal cell carcinoma, first-line therapy, tyrosine kinase inhibitors, immune checkpoints inhibitors, treatment algorithm

## Abstract

The treatment landscape for metastatic renal cell carcinoma (mRCC) has undergone significant transformations in recent years. The introduction of novel combination therapies involving tyrosine kinase inhibitors (TKI) and immune checkpoint inhibitors has resulted in improved oncological outcomes compared to traditional TKI monotherapy. In this evolving paradigm, the pivotal role of the multidisciplinary tumor board is underscored, particularly in shaping the therapeutic trajectory for patients eligible for locoregional interventions like cytoreductive nephrectomy and metastasectomy. In cases where systemic treatment is deemed appropriate, the absence of direct comparisons among the various combination therapies complicates the selection of a first-line approach. The clinician is faced with the challenge of making decisions based on patient-specific factors such as performance status, risk classification according to the International Metastatic Renal Cell Carcinoma Database Consortium, comorbidities, and disease characteristics, including the number and location of metastases and tumor histology. Considering these concerns, we propose, as a member of a Tuscany Interdisciplinary Uro-Oncologic Group, an algorithm to streamline the decision-making process for mRCC patients, offering guidance to clinicians in their day-to-day clinical practice.

## 1. Introduction

Renal cell carcinoma (RCC) represents about 3% of global tumors, with a higher prevalence in Western countries (around 10 cases per 100,000) [1,2]. Primarily affecting the elderly, with a peak incidence in the eighth decade and a slight male predominance (male: female ratio 1.5:1), RCC has seen a 2% annual increase in incidence globally and in Europe [3].

The etiology of RCC is mostly unknown, with age identified as a primary risk factor and additional factors contributing mainly tobacco smoke, obesity, and diabetes [2,3,4,5,6]. Genetic syndromes, particularly Von Hippel Lindau syndrome (VHL), play a role, with a specific autosomal dominant mutation in the VHL gene increasing the risk of kidney tumor development to around 70% by age 60 [7]. A familial history of kidney tumor diagnosis doubles the risk of RCC development [8].

RCCs include various histopathologic entities classified by the World Health Organization [9]. Histological differences indicate cytological and genetic distinctions, with characteristics like histotype, nuclear grade, and the presence of a sarcomatoid component holding prognostic value. The main RCC types, clear cell (ccRCC), papillary (pRCC), and chromophobe (chRCC), make up 65–70%, 15–20%, and 5–7% of all RCCs, respectively [10]. Clear cell RCC exhibits a significantly worse 5-year survival (43–83%) compared to pRCC (61–90%) and chRCC (80–100%) histotypes [11]. Each of these histological variants may present with a rhabdoid or sarcomatoid differentiation. Tumors with a rhabdoid and/or sarcomatoid component are classified as grade 4, indicating dedifferentiation and serving as a negative prognostic factor [12,13].

Over 50% of RCCs are incidentally detected early, allowing for curative surgical intervention. However, around 20–30% of patients are diagnosed with metastatic disease from the beginning. Clinicians use the Memorial Sloan Kettering Cancer Center (MSKCC) [14] and International Metastatic Renal Cell Carcinoma Database Consortium (IMDC) [15] risk scores to assess prognosis and guide treatment. Patients are stratified into three prognostic categories based on the number of risk factors: favorable, intermediate, and poor [14,15].

The management of mRCC has undergone significant changes, with innovative combination therapies showing better outcomes compared to traditional monotherapy [16]. The multidisciplinary tumor board plays a crucial role, especially for patients eligible for local interventions. In situations where systemic treatment is considered, the lack of direct comparisons among combination therapies complicates the choice of a first-line approach. Clinicians face the challenge of decision making based on patient-specific factors, including performance status (PS), IMDC risk classification, comorbidities, and disease characteristics such as the number and location of metastases and tumor histology. In response to these challenges, we propose an algorithm to streamline the decision-making process for mRCC patients, offering guidance to clinicians in their daily practice.

## 2. Advanced RCC Management

In RCC treatment, we distinguish localized disease and advanced disease settings. Surgical resection remains the only potentially curative approach, but it represents the standard of care only in localized forms. Conversely, in advanced disease (both at diagnosis and in the event of unresectable relapses), the main treatment is represented by systemic therapies.

### 2.1. Active Surveillance

A subset of metastatic RCC patients exhibits indolent growth. Due to the toxicity and noncurative nature of systemic therapies, certain individuals may benefit from initial active surveillance (AS) [17,18,19,20]. AS involves monitoring tumor evolution through serial imaging (ultrasound, computed tomography, or magnetic resonance imaging), with intervention reserved only for significant clinical progression [21].

The role of AS is supported by a prospective phase II trial in treatment-naïve, asymptomatic, metastatic RCC patients. The trial demonstrated that a subset of patients can be observed before starting systemic therapy. Ideal candidates for this approach have fewer adverse prognostic features and limited organ sites of metastases, particularly lung metastases <2 cm or isolated lymph node metastases [22]. Patients with sub centimeter pulmonary nodules are well suited for surveillance, as they are less likely to experience disease progression [23].

Adrenal and pancreatic metastases, more common in patients with late (>5 years) relapses, may indicate an indolent disease course, with estimated overall survival exceeding 40 months [24]. Pancreatic metastases from RCC are often asymptomatic and detected during follow-up investigations after primary lesion surgery or as an incidental finding.

Independent predictive factors for rapid progression under active surveillance include KPS < 100, liver metastasis, and a shorter time from diagnosis to AS [25]. Postponing active systemic treatment can mitigate or reduce drug-related toxicities without compromising potential efficacy. Implementing an AS protocol is feasible for well-selected patients with favorable disease (per IMDC risk classification), especially those unfit for systemic treatments, where the risk of toxicity outweighs the potential treatment benefits.

### 2.2. Locoregional Approaches

Oncology guidelines emphasize the role of surgery and local therapy in treating localized RCC. Locoregional approaches like radical nephrectomy, partial nephrectomy, radiation therapy (RT), and thermal ablation (radiofrequency ablation or cryoablation) [26] are well established for localized disease, but their standardized use in the metastatic setting is challenging.

Cytoreductive nephrectomy (CN) has a palliative role in advanced RCC, evaluated in trials like CARMENA and SURTIME [27,28]. CARMENA showed no superiority of upfront CN over sunitinib alone in asymptomatic primary tumors. SURTIME, prematurely closed, suggested delayed nephrectomy might offer benefits in terms of overall survival (mOS: 32.5 vs. 15 months—HR 0.57).

Consolidative CN is an option for local symptoms or near-complete responses to systemic therapy. However, CN is not recommended for low performance status, poor-risk IMDC class, or sarcomatoid differentiation [29].

Studies on CN in the metastatic setting often involve TKI-based systemic treatment; there is no evidence yet for ICI/TKI combination therapy. Locoregional approaches for oligometastatic or oligoprogressive disease can improve survival outcomes. Complete metastasectomy, when feasible, shows a significant mOS improvement (40.7 vs. 14.8 months) [30,31]. Recently, a system that includes tumor and patient features has been suggested for the prediction of overall mortality in mRCC patients undergoing CN (REMARCC model), with results better than IMDC and MSKCC to classify mRCC patients’ prognosis [32]. Subsequently, a subclassification of the “M” stage of mRCC into two clinical substage categories (M1 (≤3, oligometastatic) and M2 (>3, polymetastatic)) was performed based on the metastatic burden, which was correlated to distinct tumor groups whose identification improves the predictive capacity compared to the current staging [33]. Very newly, CN timing and outcomes among patients who received TKI versus IO therapy have been evaluated. Findings showed that, for patients who underwent CN, systemic therapy before CN was associated with better outcomes and that IO therapy was associated with better survival outcomes in comparison to TKI therapy [34].

With the aim of making the patient free from macroscopic disease surgery or alternative local treatments like single-beam radiotherapy (SBRT), stereotactic radiosurgery (SRS), CyberKnife RT, hypo fractionated RT, and thermal ablation can be considered after multidisciplinary discussion. Favorable outcomes are associated with good PS, solitary/oligometastases, metachronous disease (DFI > 2 years), no progression on systemic therapy, low/intermediate WHO grade, and complete resection [35]. Given the lack of general guidelines for selecting cases for local metastases treatment, patient selection should be discussed in a multidisciplinary team.

### 2.3. First-Line Treatment for Metastatic RCC

#### 2.3.1. TKI Monotherapy

Neoangiogenesis in RCC, as a result of vascular endothelial growth factor (VEGF) and platelet-derived growth factor (PDGF) overexpression due to VHL inactivation, plays a key role in the development and progression of this disease [36]. The evidence of this etiopathogenetic mechanism subsequently led to the development and study of target therapies which still represent a fundamental backbone in the management of metastatic RCC.

Sunitinib and its active metabolite (SU012662) are selective inhibitors of multiple TKI receptors associated with tumor growth and angiogenesis. The efficacy of first-line monotherapy with sunitinib has been evaluated in a randomized phase III trial [37,38], in which it has been compared to IFN-a. Median PFS was longer in those patients receiving sunitinib across all risk groups. Updated results showed a significant improvement of OS with sunitinib with respect to IFN-a in the first-line setting (mOS: 26.4 vs. 21.8 months), even though crossover from INF-a to sunitinib was permitted. Based on these data, first-line sunitinib represents a valid treatment option across all risk groups.

Pazopanib is a second-generation, multitargeted TKI active against VEGF receptor (R)-1, -2, and -3, PDGF receptor (R)-α and-β, and c-Kit. The COMPARZ trial [39], a noninferiority study of sunitinib vs. pazopanib, showed similar efficacy in terms of both PFS and OS. Based on these data, pazopanib is also a recommended regimen for patients with ccRCC across all risk groups.

Cabozantinib is a TKI active against VEGF, MET, and AXL, whose mutations seem to be correlated with acquired resistance to VEGF/VEGFR inhibitors [40].

In the open-label, randomized phase II CABOSUN trial, patients with intermediate- or poor-risk advanced RCC have been randomized to receive either cabozantinib or sunitinib. Those treated with cabozantinib showed a significantly increased median PFS (8.2 vs. 5.6 months) and higher ORR (46% vs. 18%) compared with those treated with sunitinib [41]. The incidence of grade 3 or 4 adverse events was 68% for cabozantinib and 65% for sunitinib. No significant differences have been found in terms of OS. Based on these results, cabozantinib represents a further monotherapy option for poor- and intermediate-risk patients with advanced RCC.

#### 2.3.2. Combinations of Immunotherapy and TKIs

Monotherapy with immune-checkpoint inhibitors (ICIs) directed against programmed death (PD)-1-axis or Cytotoxic T-Lymphocyte Antigen 4 (CTLA-4)-axis, instead, has been initially evaluated as the second or third line of treatment in RCC [42].

Due to the immunomodulatory effect of anti-VEGF/VEGFR, which leads to an enhancement of immune cell infiltration by normalization of tumor vasculature, the combination immune oncology (IO)/TKI may have a synergistic activity.

In the last few years, various combinations IO/IO or IO/TKI have been studied as first-line treatment in advanced RCC and four of them changed the therapeutic landscape in this setting. Table 1 reports the baseline characteristics of patients enrolled in a pivotal trial of combined IO–TKI or IO–IO.

##### Nivolumab Plus Ipilimumab (N-I)

The phase III trial Checkmate-214 compared activity and efficacy of N-I combination vs. sunitinib as first-line treatment in advanced RCC [43]. Among patients in the intention-to-treat (ITT) population, 23% had IMDC favorable-risk, 61% had intermediate-risk, and 17% had poor-risk prognostic features. About 79% of the whole population had more than two metastatic sites and lung was the most involved organ (69% of cases). At the updated 5-year follow-up analysis, the immuno-based combination demonstrated statistically significant survival advantages in both intermediate–poor (I/P) risk patients (a prespecified primary endpoint of this trial; mOS: 47.0 vs. 26.6 months with sunitinib—HR 0.68; CI: 0.58–0.81) and ITT population (a secondary endpoint; mOS 55.7 months vs. 38.4 months with sunitinib—HR 0.72; CI: 0.62–0.85) [47]. Activity outcome advantages (overall response rate (ORR) and median duration of response (mDOR)) also remained consistent and significantly in favor of N-I combination over sunitinib in I/P risk patients (42% vs. 27%—mDOR not reached (NR) vs. 19.7 months) and ITT population (39% vs. 32%—mDOR NR vs. 24.8 months), with a median time to response of 2.8 months (vs. 4.0 months with sunitinib) among ITT patients. Particularly, the IO/IO combination showed a complete response (CR) rate of 11% (vs. 2%) in the I/P risk population. Treatment-related adverse events (TRAEs) of any grade were similar between the two treatment arms (94% vs. 97.4% with sunitinib) but, considering only grade 3–5 AEs, these were found to be less frequent with the IO/IO combination (47.9% vs. 64.1% with sunitinib). Activity and efficacy outcomes in favorable-risk patients have been evaluated in an exploratory analysis: median PFS was longer (12.4 vs. 28.9 months—HR 1.84; CI: 1.29–2.62) and ORR was higher (29.6% vs. 51.6%) with sunitinib, while DOR and rate of CRs were higher (12.0% vs. 6.5%) with the IO/IO combination. No significant OS advantages emerged among favorable-risk patients, even though the HR in this subgroup has decreased over time (from 1.45 at 17.5 months minimum follow-up to 0.93 at 4-year minimum follow-up; CI: 0.64–14) and sunitinib patients spent more time on protocol treatment with toxicity, whereas N-I patients spent more time off treatment without toxicity. A subsequent analysis focusing on activity and efficacy in the presence of sarcomatoid features has been conducted [48]; in 139 I/P risk patients with ccRCC and sarcomatoid dedifferentiation included in the Checkmate-214 trial, N-I produced a significant improvement in PFS (mPFS: 26.5 vs. 5.5 months—HR 0.50; CI: 0.32–0.80), OS (mOS: 48.6 months vs. 14.2 months—HR 0.46; CI: 0.29–0.71), and ORR (60.8% vs. 23.1%); 23% of patients reached a CR at an extended 5-year minimum follow-up analysis (vs. 6.2% with sunitinib). At the genitourinary American Society of Clinical Oncology (ASCO) 2024 annual meeting, 8-year survival and safety outcomes were reported. The data demonstrated that, compared to sunitinib, treatment with N-I maintained a stable hazard ratio for OS, improved PFS in the ITT and intermediate/poor-risk patients, showed deep and durable responses with a longer duration of response and a higher number of complete responders, and continues to have manageable long-term safety [49]. Based on the above-mentioned results, N-I combination has been approved as one of the first-line treatment options in poor- and intermediate-risk patients with metastatic RCC.

##### Pembrolizumab Plus Axitinib (P-A)

The combination of pembrolizumab, an antiPD-1 antibody, plus axitinib, a VEGFR inhibitor, has been approved as first-line treatment for metastatic RCC on the basis of the Keynote-426 trial results [44]. This study compared the P-A combination with sunitinib monotherapy in treatment-naive patients with advanced RCC. The primary endpoints of this study were OS and PFS in the ITT population. The key secondary endpoint was ORR. According to the IMCD risk category, 31.2% of the study population belonged to the favorable-risk class, 56.2% to the intermediate one, and 12.5% to the poor one. About 75% of the whole population had more than two metastatic sites and lung was the most involved organ (72% of cases). Moreover, about 83% of the population had already been treated with previous nephrectomy. This study showed significant improvements in mOS (45.7 vs. 40.1 months, HR 0.73) and mPFS (15.7 vs. 11.1 months, HR 0.68) in the ITT population. At a median follow-up of 67.2 months, ORR reached 60.6% with the combination (vs. 39.6% with sunitinib) with 11.6% of CRs (vs. 4.0% with sunitinib) in the ITT population [47]. Significant advantages emerged considering the subgroup of I/P risk patients, both in terms of efficacy (mOS: 50.6 vs. 37.6 months, HR 0.64; mPFS: 13.8 vs. 8.2 months, HR 0.67) and activity (ORR 56.5% vs. 34.9%, with CRs being 9.2% vs. 2.3%). Considering the favorable-risk subgroup of patients, an advantage remained in terms of mPFS (20.7 vs. 17.8 months, HR 0.76), while mOS was similar between the two treatment arms (72.3 vs. 73.0 months, HR 1.17). The most common TRAEs were diarrhea, fatigue, hypertension, hand-foot syndrome, and hypertransaminasemia. Based on the Keynote-426 results [44], today, the P-A combination represents a treatment option that can be used as first-line treatment for metastatic RCC.

##### Nivolumab Plus Cabozantinib (N-C)

CheckMate 9ER recently led to approval of another TKI/IO combination as first-line treatment in metastatic RCC: within this trial, the combination NC showed superior PFS, OS, and ORR in comparison with sunitinib in patients with previously untreated advanced RCC [45]. Among patients in the ITT population, 22.4% had IMDC favorable-risk, 57.8% had intermediate-risk, and 19.8% had poor-risk prognostic features; 25.5% had at least 1% and 74.5% had less than 1% (or indeterminate) tumor PD-L1 expression at the time of stratification. About 80.5% of the whole population had more than two metastatic sites and lung was the most involved organ (73.7% of cases), followed by lymph nodes (40%), bone (24%), and liver (23%). When considering the ITT population, mOS reached 49.5 months in the N-C group and 35.5 months in the sunitinib group (HR 0.70—CI: 0.56–0.87), while mPFS reached 16.6 months in the combination group and 8.4 months in the control arm (HR 0.58—CI: 0.48–0.71). The I/P risk population derived the highest benefit from TKI/IO treatment (mPFS: 16.4 vs. 7.1 months, HR 0.55—CI: 0.45–0.69), while, in the favorable-risk subgroup of patients, an advantage was observed in terms of mPFS (21.4 vs. 13.9 months, HR 0.72), with no mOS difference between the two treatment arms (NR vs. 47.6 months, HR 1.07). Regarding ORR, this was higher with the TKI/IO combination in comparison with sunitinib (55.7% vs. 28.4%), with CRs of 12.4% vs. 5.2% and mDOR of 23.1 vs. 15.1 months. The most common grade 3–4 TRAEs were hypertension (13% vs. 12%), hand-foot syndrome (8% in both arms), and diarrhea (7% vs. 5%). Grade 3–4 treatment-related serious AEs occurred in 22% of patients in the N-C group vs. 10% in the sunitinib group. As said for the PA combination, the use of N-C combination is approved both for favorable risk and I/P risk RCC.

The NC combination may be preferable to TKI monotherapy in those favorable-risk situations where the patient has a high disease burden or significant symptoms or when the metastatic sites correspond to critical sites. In I/P risk patients, the TKI/IO combination is always preferable in order to obtain a more rapid response. Now, there are no direct head-to-head comparisons showing whether, in the general population or in specific subgroups, there is a significant difference between P-A and N-C combinations. An exploratory analysis of patients with liver metastases and those who have not undergone nephrectomy seemed to highlight more favorable outcomes with N-C combination in comparison with sunitinib, particularly in terms of OS (HR 0.54; CI: 0.37–0.78), PFS (HR 0.62; CI: 0.43–0.89), and ORR (41.6% vs. 23.2%) [50].

##### Pembrolizumab Plus Lenvatinib (P-L)

Lenvatinib is an oral multi-target TKI directed against VEGFR1-3, FGFR1-4, PDGFR-a, RET, and KIT; it has been evaluated as monotherapy or combined with everolimus as first-line treatment in metastatic RCC and compared with everolimus monotherapy [46].

The combination arm showed a clinically and statistically significant advantage in terms of mPFS (14.6 vs. 5.5 months) and mOS (25.5 vs. 15.4 months) when compared with everolimus alone. No statistically significant differences emerged, in contrast, in the comparison between lenvatinib monotherapy and everolimus monotherapy (HR 0.68, 95% CI 0.41–1.14; p 0.12), probably due to small sample size and imbalance in patient characteristics, resulting in a higher proportion of patients with more than three disease sites in the lenvatinib-only arm [51].

The CLEAR study is a randomized phase III trial that evaluated efficacy of two experimental treatment arms (lenvatinib plus everolimus or LP) vs. sunitinib monotherapy as first-line treatment in advanced RCC. The primary end point of the study was PFS; OS and ORR were key secondary end points. According to the IMDC prognostic risk group, about 32.5% of the patients were favorable-risk, 55.8% were intermediate-risk, and 10.4% were poor-risk; 6.8% of the whole population presented sarcomatoid features. A proportion of 68.2% of the patients presented two or more metastatic sites. The most common involved organ was lung (68.5%), followed by lymph nodes (46%), bone (25%), and liver (12.1%).

Patients were randomly assigned in a 1:1:1 ratio to receive P-L, lenvatinib plus everolimus or sunitinib.

At a median follow-up of 26.6 months, the primary endpoint of median PFS significantly favored both pembrolizumab (23.9 months, HR 0.39; CI: 0.32–0.49) and everolimus (14.7 months, HR 0.65; CI: 0.53–0.80) combinations when compared with sunitinib monotherapy (mPFS: 9.2 months); advantages in mOS emerged only for pembrolizumab combination (HR 0.72; CI: 0.55–0.93).

In addition, at the 4-year prespecified final analysis presented at the ASCO congress 2023, P-L combination maintained a statistically significant advantage in terms of OS when compared to sunitinib monotherapy, with an HR of 0.79 (CI: 0.63–0.99), but this was not clinically relevant, with an absolute advantage of less than a month (mOS: 53.7 vs. 54.3 months) and survival curves of the two treatment arms overlapping [52]. The OS analysis adjusted for subsequent treatments showed that only half of patients in the P-L treatment arm received a second line (vs. 68% in sunitinib arm); in this analysis, survival curves diverged and remained more clearly separated over time, with an HR of 0.55 (CI: 0.44–0.69) and a mOS not reached (vs. 32 months with sunitinib). The IMDC prognostic groups were not a stratification factor but, at the subgroup analysis, the P-L combination does not seem to provide advantages in terms of OS among favorable-risk patients (HR for OS: 0.94—CI: 0.58–1.52); this population may have had an impact on the global population OS results. The OS benefit is, instead, confirmed in the I/P population (HR for OS: 0.74; CI: 0.57–0.96).

Unlike OS, the PFS final analysis remains statistically significant, with a 53% reduction of progressive disease with the combination treatment (HR 0.47; CI: 0.38–0.57; mPFS: 23.9 vs. 9.2 months) and this benefit seems to be maintained at the IMDC risk subgroup analysis. P-L combination also improved ORR (71.3% vs. 36.7%) and mDOR (26.7 vs. 14.7 months; HR 0.57) when compared with sunitinib, with an increase in terms of severe AEs (74.1% vs. 60.3%).

Based on the above-mentioned results, the combination P-L has been recently approved as a further option as first-line treatment of advanced RCC.

## 3. Possible Future Perspectives: Triplets vs. Doublets

Although N-I combination demonstrated statistically significant advantages compared with sunitinib in I/P risk mRCC patients, a primary refractory rate of 20% was recorded, highlighting how certain kidney tumors seem to be strongly dependent from angiogenesis.

COSMIC-313 is a phase III randomized clinical trial evaluating N-I plus cabozantinib vs. N-I plus placebo as first-line treatment in metastatic RCC patients at I/P risk [53]. At a medium follow-up of 20.2 months, triplet therapy led to a 26% reduction in terms of disease progression when compared with doublet therapy, with a mPFS of 16.1 vs. 11.3 months (HR 0.74; CI: 0.58–0.94); this advantage was more marked in intermediate-risk patients (HR 0.68; CI: 0.540.86, with mPFS of 17.9 vs. 11.3 months). Advantages favoring the triplet therapy emerged also in terms of ORR (43% vs. 36%) and disease control rate (86% vs. 73%). The OS data are still immature and further updates in terms of efficacy and activity are needed in order to understand if this triplet may represent a new first line treatment option in metastatic RCC. However, the occurrence of an excess of G3 and G4 AE in the experimental arm (79%) compared to the control arm (56%) could represent a limitation for a possible triplet approval.

## 4. Tuscany Interdisciplinary Uro-Oncologic Group (GIOTTO) Algorithm

**Figure 1:**
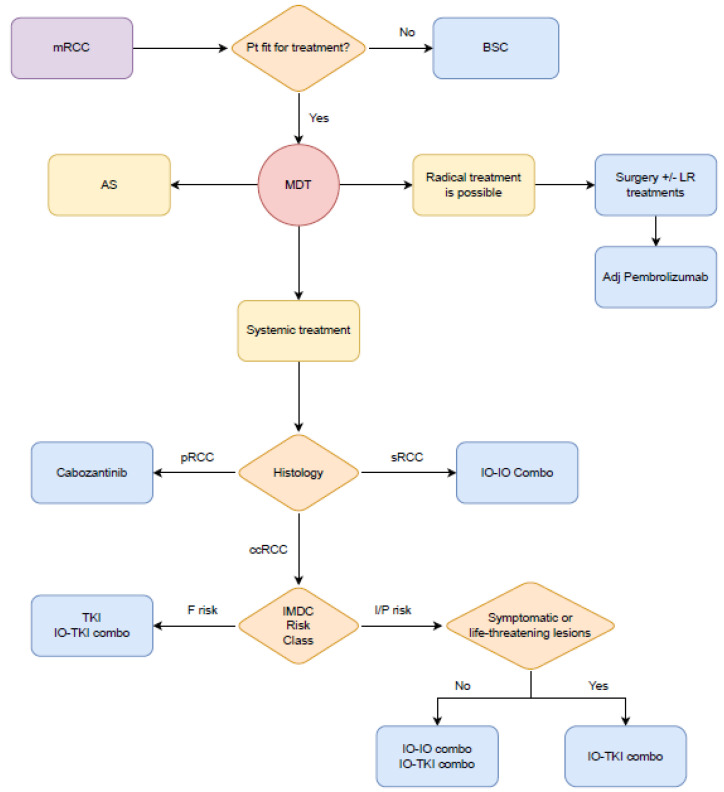
Management of metastatic renal cell carcinoma. mRCC, metastatic renal cell carcinoma; pt, patient; BSC, best supportive care; MDT, multidisciplinary team; AS, active surveillance; LR, locoregional; pRCC, papillary renal cell carcinoma; sRCC, renal cell carcinoma with sarcomatoid or rhabdoid component; ccRCC, clear cell renal cell carcinoma; IMDC, international metastatic renal cell carcinoma database consortium; TKI, tyrosine kinase inhibitor; F risk: favorable risk; I/P risk, intermediate/poor risk.

Several options exist for first-line RCC treatment today. As part of the GIOTTO working group, a team of Italian oncologists specializing in genitourinary tract pathologies from the Tuscany region, we have outlined considerations based on current evidence. These aim to aid clinicians in selecting first-line RCC treatment and are complemented by a flowchart (Figure 1) guiding clinicians through available therapeutic options. This consensus aims to ensure optimal patient treatment in our region and standardize patient management practices.

When facing advanced or metastatic RCC, the GIOTTO group prioritizes evaluating PS to determine oncological treatment feasibility over exclusive best supportive care (BSC). For eligible patients, locoregional treatments with curative intent are considered, especially after multidisciplinary discussions (MDT). MDT should be a central role in the initial phase of the therapeutic path. In mRCC, many treatment options other than systemic therapy have to be considered in most patients.

Complete disease eradication through surgery alone or in conjunction with other locoregional approaches significantly impacts the survival of patients, particularly those classified as good risk per the IMDC score, showing good PS, solitary metastases, or metachronous disease with long disease-free survival.

For patients eligible for locoregional treatments or where surgery lacks curative intent, a comprehensive evaluation for systemic treatment is crucial. In cases where nephrectomy has not been performed, consideration of CN is case-dependent, particularly for good risk patients in a multidisciplinary context. However, primary CN is not initially considered for I/P risk patients, especially with declining PS. Instead, it can be re-evaluated as a deferred option if there is a positive response to systemic treatment. CN may be considered for symptom management in certain scenarios.

Disease characteristics, including histological features and IMDC risk classification, are crucial when selecting first-line treatment. For sarcomatoid histology, combining immunotherapy with TKI or IO/IO emerges as the preferred choice. In particular, the IO/IO combo demonstrates higher benefit in terms of CRs and DOR [54].

For non-ccRCC histologies, enrollment into clinical trials should be considered as a first option [55]. If clinical trials are not available, in pRCC, on the basis of a higher level of evidence, cabozantinib should be the preferred options, even if data on IO/TKI combos are emerging [56,57]. Lenvatinib–pembrolizumab and cabozantinib–nivolumab showed in two phase II single arm trials impressive response rates but are not proven to be superior to single-agent therapy [56,57]. These combinations may be considered as alternatives to single-agent therapy [58].

For chRCC, the paucity of data does not allow a definitive conclusion whether sunitinib or everolimus and lenvatinib + everolimus or pembrolizumab represent possible options according to the ESMO guideline [58].

IMDC classification shows different benefits for combination treatments based on risk classes. TKI-IO combinations gain approval for all IMDC risk classes, while IO-IO and IO-TKI choice is pivotal for intermediate/poor-risk patients.

Considering patient characteristics, minimizing the risk of rapid disease progression is crucial for some. For those with a high disease burden, symptomatic cases, or life-threatening disease sites, TKI-IO combinations are fundamental. Both combination types can be considered for others, weighing comorbidities, pharmacological therapy interactions, and patient preferences. IO/IO combinations show the greatest benefit in OS.

For IO-TKI combinations, individual characteristics and metastatic lesion sites guide choice. Clinician experience and patient-specific factors also play a role. In favorable-risk patients, IO-TKI combinations or monotherapy may be valid alternatives, especially with a low disease burden or better prognosis sites [59,60].

For patients eligible for systemic treatments, AS may be considered in multidisciplinary discussions. Postponing systemic treatment for well-selected patients, such as those with favorable disease and limited metastases, can minimize toxicities without affecting potential efficacy.

## 5. Conclusions

Treatment of mRCC significantly changed in recent years. The new TKI/IO and IO/IO combination treatments available have improved the oncological outcomes compared to the TKI monotherapy.

In this new scenario, the multidisciplinary tumor board plays a fundamental role in defining the therapeutic path, especially in those patients who are candidates for locoregional treatments such as CN and metastasectomy.

For patients candidate to systemic treatment, the absence of a direct comparison between the various combinations makes the first-line choice challenging. Patients’ characteristics, such as the PS, the risk class according to IMDC, and the comorbidities, and the characteristics of the disease, such as the number and location of metastases and tumor histology, may help the clinician in treatment decision.

Our algorithm aims to summarize the decision-making process in mRCC patients and to guide the clinician in everyday clinical practice.

## Figures and Tables

**Table 1:** Baseline patients’ characteristics.

Study (Ref)	Treatment Arm	Median Age (Range)—Years	Male—No. (%)	IMDC Risk—No. (%)	PD-L1 Exp < 1% vs. ≥1%—No. (%)	No. of M.Organs or Sites 1 vs. ≥2—No. (%)	Site of Metastasis—No. (%)	Previous NF—No. (%)
**CheckMate-214 [43]**	Nivolumab + Ipilimumab (N = 550)	62 (26–85)	413 (75)	F:125 (23) I: 334 (61) P: 91 (17)	386 (77) vs. 113 (23)	123 (22) vs. 427 (78)	Lung: 381 (69) Lymph node: 246 (45) Bone: 112 (20) Liver: 99 (18)	453 (82)
	Sunitinib (N = 546)	62 (21–85)	395 (72)	F:124 (31.9) I: 333 (61) P: 89 (16)	376 (75) vs. 127 (25)	118 (22) vs. 427 (78)	Lung: 373 (68) Lymph node: 268 (49) Bone: 119 (22) Liver: 107 (20)	437 (80)
**Keynote-426 [44]**	Pembrolizumab + Axitinib (N = 432)	62 (55–68)	308 (71)	F: 138 (32) I: 238 (55) P: 56 (13)	165 (38) vs. 242 (56)	114 (26) vs. 315 (73)	Lung: 312 (72) Lymph node: 199 (46) Bone: 103 (24) Liver: 66 (16)	359 (83)
	Sunitinib (N = 429)	61 (53–68)	320 (75)	F: 131 (31) I: 246 (57) P: 52 (12)	156 (36) vs. 253 (59)	96 (22) vs. 331 (77)	Lung: 309 (72) Lymph node: 197 (46) Bone: 103 (24) Liver: 71 (17)	359 (84)
**CheckMate 9ER [45]**	Nivolumab + Cabozantinib (N = 323)	62 (29–90)	249 (77.1)	F: 74 (22.9) I: 188 (58.2) P: 61 (18.9)	240 (74.3) vs. 83 (25.7)	63 (19.5) vs. 259 (80.2)	Lung: 238 (73.7) Lymph node: 130 (40.2) Bone: 78 (24.1) Liver: 73 (22.6)	222 (68.7)
	Sunitinib (N = 328)	61 (28–86)	232 (70.7)	F:72 (22.0) I: 188 (57.3) P: 68 (20.7)	245(74.7) vs. 83 (25.3)	69 (21.9) vs. 256 (78.0)	Lung: 249 (75.9) Lymph node: 131 (39.9) Bone: 72 (22.0) Liver: 53 (16.2)	233(71.0)
**CLEAR [46]**	Lenvatinib + Pembrolizumab (N = 355)	64 (34–88)	255 (71.8)	F: 110 (31) I: 210 (59.2) P: 33 (9.3)	112 (31.5) vs. 107 (30.1)	97 (27.2) vs. 254 (71.5)	Lung: 249 (75.9) Lymph node: 131 (39.9) Bone: 72 (22.0) Liver: 53 (16.2)	262 (73.8)
	Lenvatinib + Everolimus (N = 257)	62 (32–86)	266 (74.5)	F: 114 (31.9) I: 195 (54.6) P: 42 (11.8)	118 (33.1) vs. 116 (32.5)	125 (35.0) vs. 229 (64.1)	Lung: 249 (70.1) Lymph node: 163(47.9) Bone: 86(23.9) Liver: 60 (17.4)	260 (72.8)
	Sunitinib (N = 257)	61 (29–82)	275 (77.0)	F: 124 (34.7) I: 192 (53.8) P: 37 (10.4)	108 (30.3) vs. 246 (68.9)	108 (30.3) vs. 246 (68.9)	Lung: 239 (66.9) Lymph node: 159 (44.5) Bone: 97 (27.2) Liver: 61 (17.1)	275 (77)

IMDC, International Metastatic RCC Database Consortium; PD-L1, programmed death ligand 1; m. metastatic, no, number; NF, nephrectomy F, favorable risk; I, intermediate risk; P, poor risk.
